# Triple Therapy for Cystic Fibrosis (Elexacaftor, Tezacaftor, and Ivacaftor): Desensitization After Skin Rash

**DOI:** 10.7759/cureus.46228

**Published:** 2023-09-29

**Authors:** Ana Isabel Santos, Joana Pacheco, Jessica Cemlyn-Jones, Fernanda Gamboa

**Affiliations:** 1 Pulmonology Department, Hospital and University Centre of Coimbra, Coimbra, PRT

**Keywords:** cystic fibrosis triple therapy, adverse drug effect, drug-induced urticarial rash, desensitization treatment, cftr modulator, cystic fibrosis (cf)

## Abstract

Cystic fibrosis (CF) is an autosomal recessive disorder of the CF transmembrane conductance regulator (CFTR) gene. CFTR modulators are novel approved therapies, and triple therapy with elexacaftor/tezacaftor/ivacaftor (ELX/TEZ/IVA) is the current gold standard for patients with at least one F508del mutation. CFTR modulators are usually well-tolerated, but some adverse effects may occur, including skin rash.

We report a case of a female patient who developed a severe skin rash after initiating treatment with ELX/TEZ/IVA. Modulator therapy and contraception were discontinued, and consequently, there was a drop in lung function and reappearance of respiratory symptoms. After rash resolution, a gradual reintroduction of ELX/TEZ/IVA was started, and this is the protocol the authors have summarized.

Triple therapy with CFTR modulators has a significant impact on lung function and the quality of life of CF patients who have at least one F508del mutation, justifying its reintroduction and desensitization even after a severe adverse effect.

## Introduction

Cystic fibrosis (CF) is an autosomal recessive disorder affecting the CF transmembrane conductance regulator (CFTR) gene, resulting in the alteration of CFTR protein synthesis, processing, or function. CFTR modulators are novel approved therapies for the treatment of CF patients with specific CFTR mutations. Elexacaftor/tezacaftor/ivacaftor (ELX/TEZ/IVA, Kaftrio®, Vertex Pharmaceuticals, Boston, MA) was approved in Europe in August 2020 for the treatment of CF in patients aged 6 years and above and who have at least one F508del mutation. ELX and TEZ increase the number of CFTR proteins on the cell surface, while IVA improves the activity of the defective CFTR protein. Clinical trials were promising, and they showed significant improvement in lung function tests and reduction of pulmonary exacerbations, sweat chloride, and body mass index in comparison to placebo [1,2]; real-life studies are ongoing.

In general, CFTR modulators were shown to be well tolerated. The most common side effects of ELX/TEZ/IVA (which may affect more than one in 10 patients) are headache, diarrhea, an increase in liver enzymes, and upper respiratory tract infections [3]. Skin rashes may occur, being documented as a common adverse reaction (which may affect up to one in 10 patients) [3], and do not usually require drug discontinuation [1,2]. A rash is more common in women, particularly those on hormonal contraception [1,2].

The way that ELX/TEZ/IVA is reintroduced after discontinuation is not well established, and here, we report the desensitization protocol used in the case of a severe cutaneous rash reaction to ELX/TEZ/IVA. 

This article was previously presented as a meeting poster at the 38th Portuguese Society of Pneumology Congress on November 12, 2022, Vilamoura, Portugal, and as an oral communication at the 46th European Cystic Fibrosis Conference on June 23, 2023, Vienna, Austria.

## Case presentation

The case reports a 38-year-old woman diagnosed with CF at the age of 14, F508del/R334W CFTR genotype, with chronic bronchial infection by *Burkholderia cepacia* and *Pseudomonas aeruginosa*. CF symptoms were persistent intermittent productive cough with yellowish/greenish sputum, and spirometry evaluation showed an FVC of 2.96 L (80.6 %) and an FEV1 of 1.86 L (58.3%). The patient is allergic to ceftazidime, and every time she was admitted for endovenous treatment (on average once a year), it was necessary to comply with a desensitization protocol, which was drawn up by an allergy consultant.

The patient had no previous history of CFTR modulator therapy and was taking hormonal contraception. She was started on ELX/TEZ/IVA in September 2021. During the first few days, she had an unusually productive cough but rapidly improved, and gradually, she felt less breathless and also showed a decrease in cough and fatigue, with remarkable improvement in respiratory function and symptoms.

**Figure 1:**
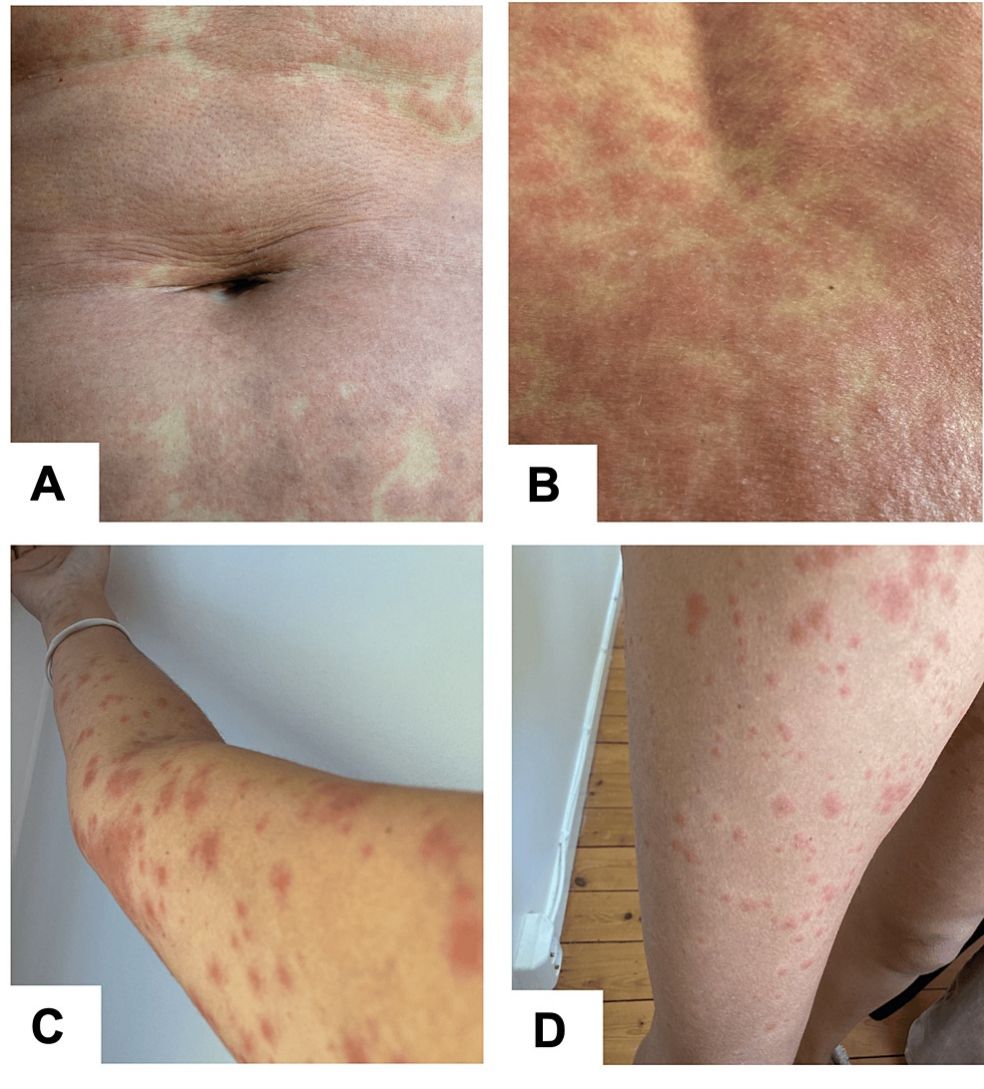
Widespread rash on patient’s (A) abdomen, (B) back, (C) arm, and (D) leg

After four days with ELX/TEZ/IVA, FVC improved by 0.18 L and FEV1 increased by 0.22 L. However, eight days later, she developed an erythematous rash (Naranjo Adverse Drug Reaction Probability Scale = 9 [4]), which was initially located in the abdomen and lower back but gradually became widespread, with the involvement of arms, legs, and face (Figures 1A-1D). At the first signs of rash, both ELX/TEZ/IVA and oral contraception were discontinued, and corticosteroids (betamethasone 4 mg) and high-dose antihistamine drugs (desloratadine 5 mg bid) were started. Rash and itching persisted with only a mild improvement after a week. After eight days of discontinuing ELX/TEZ/IVA, she relapsed to low energy, fatigue, headache, and subfebrile temperature, and her sputum reappeared. There was also a decrease in lung function tests: FVC decreased by 0.20 L and FEV1 decreased by 0.13 L, and she was started on antibiotics for exacerbation.

One month later, after the complete resolution of the rash, medication was gradually reintroduced, first by taking one tablet of IVA per day. On the fifth day, approximately five hours after the first tablet of ELX/TEZ/IVA, another widespread rash occurred, and again treatment was stopped. The new rash was milder, and the resolution of it was faster. A week later, a different scheme of drug reintroduction combined with antihistamine drugs was undertaken without any side effects, as shown in Table 1. Hormonal contraception was not reintroduced.

**Table 1:** Desensitization protocol for ELX/TEZ/IVA reintroduction ELX, elexacaftor; IVA, ivacaftor; TEZ, tezacaftor

Duration	Takes	ELX/TEZ/IVA morning	IVA evening
2 weeks	Each 4 days	1 tablet	0
1.5 weeks	Each 3 days	1 tablet	0
1 week	Each 2 days	1 tablet	0
5 days	Daily	1 tablet	0
5 days	Daily	1 tablet	1 tablet
1 week	Every other day	1 tablet/2 tablets	1 tablet
Until now	Daily	2 tablets	1 tablet

## Discussion

Triple therapy consists, in a daily dose, of two tablets containing ELX 100 mg/TEZ 50 mg/IVA 75 mg in the morning and one tablet containing IVA 150 mg in the evening. In our patient, IVA does not seem to be the responsible agent for the adverse reaction because there was no recurrence of the rash when single therapy with IVA was reintroduced. However, it was not possible to perceive whether ELX or TEZ was accountable for the reappearance of the rash after taking the triple association tablet.

When in the presence of a severe treatment-induced rash, medication should be immediately discontinued. The mechanism behind this immunological adverse event is not clear, and there is a single case report of a patient with ELX/TEZ/IVA-induced rash that resolved without drug discontinuation [5].

On drug reintroduction, ELX/TEZ/IVA dose should be carefully titrated, with a slow and gradual increase, in order to achieve desensitization and tolerance to allow dose escalation. In this particular case, the protocol of reintroduction of full dose took almost two months. A few cases of skin rash following the introduction of triple therapy for CF were published, and the desensitization protocol for reintroduction is not clear.

The up-to-date published desensitization protocols refer to different circumstances, and reintroduction approaches were different when compared to our patient. In a report of two pediatric patients with an ELX/TEZ/IVA drug-induced rash, the extension of the rash was lower, and the titration protocol had a duration of 23 days, starting with the introduction of a minor dose of IVA alone [6]. In another two reports described in one case, there was an approach for desensitization after a severe rash in an adult male patient started with one-eighth of a tablet of ELX/TEZ/IVA, which was gradually increased and well tolerated [7]. The other report described two adult female patients under hormonal contraception, in which one of them had previous exposure to CFTR modulators that developed a skin rash leading to interruption of contraception and CF triple therapy. In this case, there was a successful eight-week titration protocol with the reintroduction of morning and evening tablets simultaneously [8].

In conclusion, due to the heterogeneity of rash presentation, it is unlikely to find a “one size fits all” protocol for desensitization of every cutaneous adverse reaction of ELX/TEZ/IVA, and it is important to be aware and have access to different titration schemes in order to achieve a successful reintroduction of this life-changing therapy.

## Conclusions

As ELX/TEZ/IVA is the current gold standard of treatment for the majority of patients with CF, health professionals must be aware of common side effects, and it is important to establish and make available desensitization protocols to manage these side effects. Several desensitization protocols can be used, and here, we present the one that worked successfully with our patient. As patients strongly benefit from treatment with ELX/TEZ/IVA and in the absence of effective alternatives, the reintroduction of the same drugs with the risk of rash recurrence is acceptable, given the benefits in the quality of life of patients with CF.
